# Case of Retention of Urine, Produced by Spasmodic Stricture, Effectually Relieved by Leeching

**Published:** 1822-12

**Authors:** R. Price


ART. V.—*Case of Retention of Urine, produced by Spasmodic Stricture, effectually relieved by Leeching.* By R. PRICE, Esq.

MR. B—, ætat. thirty-seven years, applied to me, early in the morning of the 8th of July last, in a case of retention of urine, under which he had laboured since the afternoon of the preceding day. He appeared to suffer extreme pain. On questioning him, I found he had not been subject to any such affection before; neither could he assign any cause for the present attack.

I immediately attempted the introduction of a moderate-sized catheter, but found myself unable to accomplish it, in consequence of the existence of a spasmodic stricture, about the commencement of the varicous portion of the urethra. I successively tried catheters and metallic instruments of various sizes, but, after much perseverance, was foiled in all my attempts. I then directed the abstraction of twenty-four ounces of blood from the arm; after which, I again endeavoured to introduce an instrument, but with no better success. Ultimately, however, I succeeded in passing a small metallic sound, about the size of a knitting-needle; but, on withdrawing it, no urine followed. I prescribed—

℞. Hydrargyri Submur.
Extract. Colocynth c. āā ℈ss.
Ol. Caryophil. gtt. j.
Syrup. q. s. fiat pilul. iv. statim capienda c. haust. sequent.

℞. Ol. Ricini, ℥jss.

Tincturæ Sennæ, ℥ss.

Tincturæ Opii, min. xxx. M. fiat haustus.

and directed him to return home, and to sit for some time in a hot hip-bath.

I visited him in the evening at his residence in the Borough, and, having to pass the house of my friend, Mr. Robert Rowley, surgeon, (a gentleman who is estimated particularly expert at the introduction of the catheter,) I requested the favour of him to accompany me. Mr. Rowley attempted to introduce a metallic catheter, but, after persevering for some time, he also found his efforts unavailing. I then directed a dozen leeches to be applied immediately over the seat of the stricture, and the subsequent bleeding from their perforations to be freely encouraged with hot water; and administered forty drops of laudanum, the purgative medicines having operated freely. We saw him again early the next morning. Eight of the leeches only had performed their functions, but the subsequent hemorrhage had been very copious; from which so much benefit resulted, that, in about an hour after the application of the leeches, he voided between two and three pints of urine. The catheter was now introduced without difficulty, and the bladder completely emptied. After this he continued to discharge his urine freely, the aid of an instrument being no longer necessary, and has never since been subject to any symptoms of stricture. Mr. Rowley expressed no less surprise than pleasure at the novelty and success of the practice.

*Cannon-street, London; Oct.* 10, 1822.

