# Errata in Last Number

**Published:** 1823-01

**Authors:** 


Errata *in last Number.*

Case of Retention of Urine, p. 482, for *varicous* read *mucous.*
for *estimated* read *esteemed.*

